# Viral and Nonviral Engineering of Natural Killer Cells as Emerging Adoptive Cancer Immunotherapies

**DOI:** 10.1155/2018/4054815

**Published:** 2018-09-17

**Authors:** Sandro Matosevic

**Affiliations:** ^1^Purdue University, Department of Industrial and Physical Pharmacy, West Lafayette, IN 47907, USA; ^2^Center for Cancer Research, Purdue University, West Lafayette, IN 47907, USA

## Abstract

Natural killer (NK) cells are powerful immune effectors whose antitumor activity is regulated through a sophisticated network of activating and inhibitory receptors. As effectors of cancer immunotherapy, NK cells are attractive as they do not attack healthy self-tissues nor do they induce T cell-driven inflammatory cytokine storm, enabling their use as allogeneic adoptive cellular therapies. Clinical responses to adoptive NK-based immunotherapy have been thwarted, however, by the profound immunosuppression induced by the tumor microenvironment, particularly severe in the context of solid tumors. In addition, the short postinfusion persistence of NK cells *in vivo* has limited their clinical efficacy. Enhancing the antitumor immunity of NK cells through genetic engineering has been fueled by the promise that impaired cytotoxic functionality can be restored or augmented with the use of synthetic genetic approaches. Alongside expressing chimeric antigen receptors to overcome immune escape by cancer cells, enhance their recognition, and mediate their killing, NK cells have been genetically modified to enhance their persistence *in vivo* by the expression of cytokines such as IL-15, avoid functional and metabolic tumor microenvironment suppression, or improve their homing ability, enabling enhanced targeting of solid tumors. However, NK cells are notoriously adverse to endogenous gene uptake, resulting in low gene uptake and transgene expression with many vector systems. Though viral vectors have achieved the highest gene transfer efficiencies with NK cells, nonviral vectors and gene transfer approaches—electroporation, lipofection, nanoparticles, and trogocytosis—are emerging. And while the use of NK cell lines has achieved improved gene transfer efficiencies particularly with viral vectors, challenges with primary NK cells remain. Here, we discuss the genetic engineering of NK cells as they relate to NK immunobiology within the context of cancer immunotherapy, highlighting the most recent breakthroughs in viral vectors and nonviral approaches aimed at genetic reprogramming of NK cells for improved adoptive immunotherapy of cancer, and, finally, address their clinical status.

## 1. Introduction

Natural killer (NK) cells are part of the innate immune response against tumors and are emerging as powerful effectors of cancer immunotherapy. NK cells express a fixed set of germ line-encoded activating and inhibitory receptors, upon which they rely on for the recognition of cancer cells [1]. These receptors enable them to recognize major histocompatibility complex (MHC) class I molecules on target cells and allow them to maintain tolerance to self-tissues [2]. This is in contrast to adaptive immune cells such as T cells, which undergo receptor rearrangement to modulate target recognition. The majority of NK cells, as well as some T cells, express the receptor family natural killer group 2 (NKG2), which includes NKG2A, B, C, D, E, F, and H. Among these, NKG2A and B are inhibitory receptors. Human NK cells are typically characterized as CD3^−^CD56^+^ and differ in functionality and maturation status. The responsiveness of NK cells to tumor targets is determined by their education status [3], which ultimately regulates the level of antitumor effector function and control alloreactivity.

Despite their potent antitumor function, the pathogenesis of many cancers induces inhibition of NK cell effector function via mechanisms that include severe immunosuppression via immunometabolic and antigen escape routes [4, 5]. For those reasons, for the past decade, scientists have pursued approaches aimed at enhancing NK cells' antitumor activity and priming them to avoid immunosuppression through genetic engineering. These approaches have ranged from enhancing the proliferation of the cells following adoptive transfer via the expression of endogenous cytokines to suppression of tumor microenvironment (TME) inhibitory signals, or the enhancement of the cells' cytotoxic function. The latter approach has primarily relied on redirecting NK cells by chimeric antigen receptors (CARs). These are recombinant constructs consisting of an extracellular single-chain variable fragment (scFv) linked to intracellular signaling domains. The scFv mediates antigen recognition and binding by recognizing antigen expression on cancer cells and triggering NK cell activation [6]. Engineering of NK cells has been achieved using both viral and nonviral approaches, each defined by a set of challenges. These approaches have resulted in remarkable preclinical discoveries, though only a handful of studies have advanced through the clinical pipeline. Here, we discuss the latest advances in physical approaches for the genetic engineering of NK cells and the molecular targets used to effect their function.

## 2. NK Cell Biology Relevant to Immunotherapy

**Figure 1:**
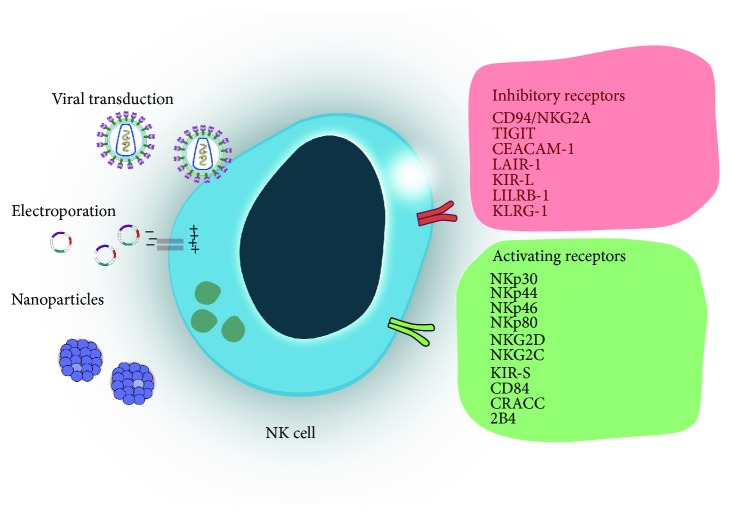
Diagram showing the three main approaches aimed at genetically engineering NK cells: viral transduction, electroporation (nonviral), and nanoparticle-based transduction (nonviral). These engineering approaches aim to enhance safety, improve cytotoxicity, and increase persistence of NK cells in the tumor microenvironment. NK cells respond to tumor targets by calibrating an array of inhibitory and activating receptors, which can be used in genetic engineering approaches to further direct NK cell function.

The cytotoxicity of natural killer cells is determined by a signaling interplay of a vast repertoire of inhibitory and activating receptors (Figure 1). Unlike T cells, NK cells do not express specific antigen receptors and do not require prior sensitization to trigger killing of target cells [7]. However, recent reports have advanced the notion that NK cells possess features of an adaptive immune response and that their cytotoxicity is most fully realized following priming by myeloid lineage cells, such as dendritic cells [8]. Understanding NK cell biology, their effector function, and their functional and metabolic interactions with the TME are key to developing targets for NK cell-based adoptive immunotherapies. The two major populations of NK cells are CD56^dim^ and CD56^bright^ NK cells, found in similar proportions in cord blood and peripheral blood [9]. Phenotypically, human NK cells are characterized by the expression of CD56 (N-CAM) and CD16 (Fc*γ*RIIIA) markers and the lack of CD3. CD56^bright^ NK cells are CD16 dim or negative, while CD56^dim^ cells are also characterized by CD16^high^ expression and represent the major circulating NK subset [10]. CD56^dim^ NK cells exert higher natural cytotoxicity, while CD56^bright^ cells―generally considered to be the precursor to the CD56^dim^ subset [11]―have higher capacity for producing cytokines [12]. Their differential expression of IL-2R is associated with differences in both subsets' proliferative capacity [13]. In addition, defined subsets of mature NK cells with memory-like features are also characterized by expression of CD57 [14] or CD62L [15], specific for CD56^dim^ NK cells. Mouse NK cells do not express CD56; however, mouse NK cells expressing the chemokine receptor CXCR3 were recently described as representing the murine equivalent to human CD56^bright^ cells [16]. It is also important to note that differentiation of NK cells into mature, functional populations is accompanied by acquisition of distinct phenotypic markers [17]―as a consequence, targeting NK cells for immunotherapies requires a precise understanding of the molecular markers of functional maturation of these cells.

Broadly, the function of NK cells is mediated by soluble factors, including chemokines, cytokines, and other secreted ligands of NK cell receptors. A single NK cell typically expresses two to four inhibitory receptors in addition to a number of activating receptors, yielding a vastly heterogeneous cell population. Through these receptors, NK cells mediate their cytotoxicity against virus-infected cells. The use of genetically engineered NK cells against tumors has relied on the ability to mediate the cells' cytotoxicity through the modulation of activating and inhibitory signals received from multiple germ line-encoded receptors [18].

Two main hypotheses have been suggested to describe NK cell activation based on their receptor profile: “missing self” and “induced self.” [19] Based on the “missing self” theory, NK cells can recognize aberrant target cells which lack inhibitory MHC molecules, in turn promoting their lysis via engagement of NK cells via activating receptors [20]. Functional control is further mediated by normal cells, which regularly express MHC and lack activating receptors. The “missing self” hypothesis is supported by a high number of studies that have shown that decreased expression of MHC class I molecules on tumor cells correlates to their higher susceptibility to NK cell killing. The complementary “induced self” mechanism of activation states that cancer cells display elevated expression of ligands for NK cell receptors such as NKG2D due to stress factors, resulting in the engagement of NK cells [21]. Despite the expression of inhibitory receptors, activation by “induced self” is able to override inhibitory signals present on cancer cells. The two mechanisms are not contradictory and likely work together to modulate the overall responses of NK cells to pathogens.

Activating receptors include the natural cytotoxicity receptors (NKp46, NKp44, and NKp30), C-type lectin-like receptors (NKG2D and CD94-NKG2C), and Ig-like receptors (2B4). Inhibitory receptors, on the other hand, include the killer immunoglobulin-like receptors (KIR) or Ig-like receptors (CD158), the C-type lectin receptors (CD94-NKG2A), and leukocyte inhibitory receptors (LIR1 and LAIR-1). Each of these receptors is associated with distinct signaling molecules: [22] NKp46, for instance, is associated with the FcR *γ* chain or the TCR *ζ* chain. NKp44 associates with the immunoreceptor tyrosine-based activation motif- (ITAM-) bearing signaling molecule DAP12, while NKG2D associates with DAP12 or DAP10, which signals through phosphatidylinositol-3 kinase and other pathways [23]. An alternative activation/inhibition pathway is facilitated by costimulatory receptors such as CD244 (2B4), member of the signaling lymphocytic activation molecule (SLAM) family, which signals through recruitment of Src homology 2 domain containing adapter proteins SAP or ERT [24]. In addition, NK cells can induce target cell death using tumor necrosis factor-*α* (TNF-*α*), Fas ligand, and TNF-related apoptosis-inducing ligand (TRAIL) [25].

Inhibition of NK cell cytotoxicity is directed by recognition of major histocompatibility complex class I molecules (MHC-I) expressed on target cells. Inhibitory KIRs signal through intracellular immunoreceptor tyrosine-based inhibitory motifs (ITIMs) [26]. Interactions between MHC molecules―human leukocyte antigens (HLAs), specifically HLA-A, HLA-B, and HLA-C―and KIR receptors [27] contribute to driving NK cytotoxicity by inducing the spontaneous killing of targets that either lack self MHC or express allogeneic MHC molecules [28]. There are no rules which dictate which KIRs express on which NK cells, though their expression is regulated by methylation of KIR gene loci [29]. Among KIR genes, three are common to all haplotypes―KIR3DL3, KIR2DL4, and KIR3DL2.

NK cell maturation is accompanied by phenotypic changes and alteration in functional potential. Less mature and differentiated NK cells are more sensitive to cytokine stimulation and respond by more potently expressing IFN-*γ*. Conversely, more mature differentiated NK cells show increased CD57 expression, a decrease in CD94/NKG2A expression, and higher KIR numbers [15]. Similarly, activation of NK cells in response to receptors on cancer cells is associated with a number of functional changes [30]. Signaling cascades following activation on NK cells are different for the various receptors [31]. Collectively, these activation mechanisms fuel the killing of target cells by NK cells via the polarized release of the contents of lytic granules at the immunological synapse. This occurs in two steps: polarization and degranulation. Firstly, microtubule organizing center (MTOC) and MTOC-associated granules traffic toward locations on the plasma membrane that are in contact with cancer cells (polarization), which is followed by fusion of the granules with the plasma membrane (degranulation) [18]. These two processes can occur independently. Polarization was shown to be transient and highly sensitive to inhibition, unlike degranulation, and occurs differently based on which cytokines are used to prime NK cells [32]. Among receptors, NKG2D and LFA-1 are able to autonomously signal for granule polarization.

Activating receptors on NK cells can also be altered due to the tumor microenvironment. Transforming growth factor-*β* (TGF-*β*), an immunosuppressive cytokine enriched in many advanced cancers, was shown to induce downregulation of NKG2D/DAP10 on NK cells [33]. Similarly, hypoxia, associated with the pathology of many solid tumors, was reported to downregulate as number of activating NK cell receptors, including NKp46, NKp30, NKp44, and NKG2D, irregardless of whether NK cells were primed with IL-2, IL-12, IL-15, or IL-21 [34]. This highlights that targeting dysregulated pathways in cancer is a meaningful approach to restore inhibition of activation signals that mediate cytotoxicity of NK cells against cancer cells.

Priming with cytokines, however, is critical for adoptive NK cell immunotherapy, as these cells cannot persist without activation. NK cells have been primed with IL-2, IL-12, IL-15, IL-18, or IL-21. Though IL-2 has been the most commonly used cytokine, the use of IL-12 and IL-15 has been shown to induce higher amounts of IFN-*γ* and thus potentiate the cells' cytotoxicity. The roles of IL-15 on NK cell homeostasis, proliferation, cytokine production, and cytotoxicity have been extensively described [35, 36]. Boieri et al. reported that preactivation of NK cells with a combination of IL-12, IL-15, and IL-18 resulted in significant enhancement of NK cell cytotoxicity and upregulation of activation markers. More importantly, adoptive transfer of these cells dramatically slowed progression of Roser leukemia *in vivo* [37].

Though priming with cytokines has been traditionally employed to activate NK cells, the highest NK expansion rates have been obtained with the use of feeder cell lines, such as HFWT, genetically modified K562 cells to express IL-15 or IL-21, and EBV-transformed lymphoblastoid cell lines (EBV-LCL). K562 cells modified to express IL-12, IL-2, and 4-1BB have yielded expansion rates of 1000-fold [38]. Expansion rates and characteristics of other feeder cell lines used for NK cell therapy have been described in detail elsewhere [39].

NK cells have been demonstrated to be promising in allogeneic adoptive transfer settings. However, prior immune suppression is required to mitigate immune reactivity of NK cell infusions. Typically, this includes a nonmyeloablative conditioning regimen using cyclophosphamide and fludarabine [40]. Recently, Curti et al. reported results of a phase I trial using KIR ligand-mismatched haploidentical NK cells to treat seventeen acute myeloid leukemia patients in complete remission after fludarabine/cyclophosphamide chemotherapy and followed by administration of IL-2. 22.5 months after allogeneic NK cell transplantation, 9/16 (56%) remained alive and disease-free. These responses correlated with the infusion of higher doses of alloreactive NK cells [41]. In recent years, emerging results from phase I and phase II trials have demonstrated safety and efficacy of allogeneic infusions of NK cells for immunotherapy of hematological malignancies and solid tumors [42].

## 3. Sources of NK Cells for Genetic Engineering

NK cells used in immunotherapy can be derived from various sources, including cord blood [43, 44], peripheral blood [45], adult hematopoietic stem cells (HSCs) [46], embryonic stem cells (ESCs) [47], or induced pluripotent stem cells (iPSCs) [48]. Most adoptive NK cell-based products are typically generated with cells enriched from peripheral blood of haploidentical donors collected by apheresis. Such collections can be performed in a closed system under cGMP conditions, thus minimizing the risk of contamination. While peripheral blood contains approximately 0.08–0.43 × 10^6^ NK cells/ml, the vast majority (about 90%) of peripheral blood NK cells are CD56^dim^CD16^+^ cells. These cells have been engineered with a wide variety of CARs targeting CD19, CD20, and ErbB2 or containing NKG2D [49]. Because obtaining sufficient numbers of NK cells for therapy is a major challenge in the preparation of these cells for clinical use, multiple approaches aimed at improving their *ex vivo* expandability have been developed [50]. Among these, the use of feeder cells is common―cocultivation of NK cells in the presence of another cell type that provides a stimulatory signal. Cells used as feeder layers have included cancer cells [51] such as the Jurkat subline KL-1 [52], genetically modified K562 cells engineered to express membrane-bound IL-15 and IL-21 fused to 4-1BB [53, 54], Epstein-Barr virus-transformed lymphoblastoid cells [55], or irradiated peripheral blood mononuclear cells in the presence of anti-CD16 antibody [56]. Particle-based approaches, such as the use of plasma membrane vesicles derived from K562-mbIL15-41BBL or K562-mbIL21-41BBL feeder cells, have also been described [57] and are currently in preclinical development.

However, multiple factors affect the number of cells that can be retrieved from peripheral blood [58]. The proportion of mature NK cells is lower in peripheral blood associated with pathological settings―Mamessier et al. [59] characterized NK cells in the peripheral blood of breast cancer patients, showing inhibition of NK cell maturation and diminished cytotoxicity, alongside a higher proportion of CD56^dim^CD16^−^ and CD56^bright^CD16^−^ cells. A similar observation was also made in a separate study looking at peripheral blood NK cells from patients with colorectal cancer [60]. These cells also exhibited deficiencies in the production of interferon-*γ* and cytotoxic granules.

While genetic modifications of peripheral blood-derived NK cells have been carried out to successfully generate functionally competent NK cells, they are nonetheless considered difficult. An alternative source of NK cells to peripheral blood is cord blood. The expansion of clinical numbers of NK cells (2 × 10^9^) from umbilical cord blood was achieved in a cGMP-compliant closed system without the need for feeder cells [61]. Benefits to using cord blood as a source of NK cells include its relative ease of collection [62], the fact that cord blood contains fewer T cells thus minimizing the risk of GvHD [63], and the presence of unique NK progenitor cells that are absent in peripheral blood [64, 65]. Cord blood NK cells have been genetically engineered to express a number of CARs: NK cells expressing antiCD19-CD28-CD3*ζ* CARs, IL-15, and iCasp9 [66] are currently in clinical trials (NCT03056339), while a similar expression system redirected against CS1 is being studied preclinically.

Both hESC- and iPSC-derived NK cells have demonstrated potent *in vivo* antitumor activity [67, 68]. hESC lack contaminating T or B cells, significantly facilitating NK cell selection, and it has been argued that they are more cytotoxic and functionally mature than umbilical cord blood-derived NK cells. In addition, they have higher levels of KIR expression than umbilical cord blood NK cells [69]. They were also found to express activating and inhibitory NK receptors similarly to peripheral blood-derived NK cells, including NKG2D, NKp46, Fas, TRAIL, and KIRs [70]. Knorr et al. [71] imaged trafficking of hESC-derived NK cells using mouse embryonic fibroblast (MEF) feeder cells to tumor sites and observed persistence of these cells for up to 25 days. Clinical scale expansion of hESC- and iPSC-derived NK cells has also been successfully demonstrated [48]. However, efforts at using these cells are largely focused on improving culture conditions―feeder lines are needed for many of the expansion protocols. Increasingly, these cells are being used in immunotherapeutic interventions. Recently, Hermanson et al. [72] showed that iPSC-derived NK cells mediate ovarian cancer killing at least as well as peripheral blood-derived NK cells. The same lab also generated NK cells from pluripotent stem cells engineered to express CD16a, which remained resistant to shedding by ADAM17 [58]. Due to the relative ease of genetically engineering pluripotent stem cells compared to NK cells from peripheral or umbilical cord blood, this example may represent a promising strategy for generating genetically modified NK cells for use in immunotherapy.

Several NK cell lines also exist. These cell lines were developed to overcome some of the difficulties with obtaining and expanding sufficient cells from blood sources. NK cell lines include NK-92, NKG, NKL, KHYG-1, YT, NK-YS, SNK-6, HANK-1, IMC-1, YTS, and NKL cells [73]. Though cell lines such as YTS have been successfully engineered with CARs signaled through protein DAP12 [74], the NK-92 cell line is the most widely studied and the only one that has shown consistent cytotoxicity against tumor targets. It is also the only NK line that has been investigated clinically. Similarly to blood-derived NK cells, NK-92 cells express CD56 and lack CD3 but unlike blood NK cells, NK-92 cells do not express KIRs and lack some activating receptors such as NKp44 and NKp46. They also lack CD16 and are thus unable to participate in antibody-dependent cell-mediated cytotoxicity (ADCC). Derived from the peripheral blood of a patient with non-Hodgkin's lymphoma, the cell line was developed by NantKwest and has undergone extensive preclinical and clinical development [75]. In a recent clinical study involving fifteen patients with either solid tumors or lymphoma/leukemia, infusion of 10^10^ NK-92 cells/m^2^ resulted in antitumor response in three-fourths of patients with lung cancer [76]. Other clinical studies with the NK-92 cell line on solid tumors have also recorded positive outcomes [77]. In addition to being used as adoptive immunotherapies, NK-92 cells have also been successfully engineered as CAR carriers [78–80]. One potential drawback to the use of the NK-92 cell line is the need for irradiation to avoid allogeneic tumor engraftment.

In summary, peripheral blood remains the most common single source of NK cells for preclinical and clinical development. However, issues of donor variability, sourcing constraints, and the inability to facilitate allogeneic therapy have fueled the search for alternative sources of NK cells that can avoid some of these drawbacks. Though cord blood-derived, engineered NK cells have progressed to clinical trials, so far, however, these cells have been engineered to target a limited number of cancer antigens and less is known about their functional and cytotoxic profile. As the field seeks routes to allogeneic therapy, alternative sources of NK cells are likely to become more prominent. The use of cell lines is unlikely to move beyond NK-92 cells, and their lack of ADCC limits their use in combination immunotherapies with monoclonal antibodies. It is likely that induced pluripotent stem cells will emerge as more prominent sources of allogeneic stem cells potentially contrasting the use of cell lines once more protocols are developed. Our knowledge of NK cell biology is constrained by a limited understanding of the role of immunometabolism on NK cell effector function: TME immunosuppression evades recognition by mechanisms other than antigen escape, and it will be important to define how NK cell maturation, licensing, memory, and education are affected by immunometabolic signaling in order to design effective therapies for targets that have been more evasive, such as solid tumors.

## 4. Engineered T *vs* NK Cells

Despite the extensive use of NK cells as immunotherapies against solid refractive tumors, T cells have so far received the most clinical attention, through approaches primarily involving immunological checkpoint blockade and adoptive cell transfer. The use of T cells has been additionally buoyed by the approval of the first two engineered CAR-T cell therapies by the FDA in 2017. NK cells, however, remain an attractive proposition that presents a number of potential advantages compared to T cells (Table 1). Unlike T cells, NK cells' killing of target cells is tumor-associated antigen-independent [81]. The long-term persistence of CAR T cells [82] postinfusion has been shown to cause B cell aplasia [83], requiring intravenous immunoglobulin replacement. This has resulted in approaches that attempt to reduce the persistence of CAR T cells after their “therapeutic window,” with companies such as Endocyte working in the space. In contrast, NK cells have very short response times, which range from minutes to hours, and typically require cytokine stimulation for sustained persistence *in vivo*, which is more limited compared to that of T cells. Withdrawal of cytokines was suggested as being detrimental to NK cell cytotoxicity, while their presence could induce changes in the functionality, cell shape, and activation properties of NK cells, bringing into question important consideration for appropriate protocols for ex vivo stimulation of NK cells [84]. IL-2-activated NK cells also act as serial killers, with each NK cells shown by live video microscopy to kill as many as four cells [85].

Broadly, toxicities associated with engineered T cells include those of neurological nature, cytokine release syndrome, anaphylaxis, off-target effects, and insertional mutagenesis [86].

For a long time, NK cells have been attractive due to the pervasive opinion that they do not induce graft-versus-host disease (GvHD). This was supported by a large number of studies involving adoptive transfer of NK cells into hematopoietic cell transplant recipients, which did not record GvHD induction after infusion [87–89]. Similarly, neither did adoptively transfer allogeneic haploidentical NK cells into lympho-depleted patients in nonallogeneic HCT settings [90]. Conversely, Shah et al. [38] observed the onset of GvHD in the presence of subthreshold T cell doses following adoptive transfer of donor-derived IL-15/4-1BBL-activated NK cells following HLA-matched nonmyeloablative peripheral blood stem cell transplantation. GvHD was higher in matched unrelated donor as opposed to matched sibling donor recipients and was thought to occur by enhancing T cell alloreactivity. Such conflicting results have advanced a dual promoting/suppressive model of NK cells in GvHD [91]. The GvHD promoting effect is thought to be associated with higher amounts of IFN-*γ*-producing NK cells after hematopoietic cell transplantation inducing acute GvHD [92]. On the other hand, the GvHD suppressive role is exerted by cytolysis of T and dendritic cells by NK cells, alongside their limited persistence *in vivo*. Elsewhere, Meinhardt et al. [93] discovered that the CD11b^+^ murine NK subset has the ability to provide protection against acute GVHD. Nonetheless, the NK cells' short persistence, depletion of alloreactive T cells, or antigen-presenting cells still make NK cells attractive as potential allogeneic immune effectors.

Related to NK cells' persistence is their promotion of immune reconstitution following adoptive cell transfer. A number of studies have investigated immune system reconstitution with NK cells and its relationship to the development of GvHD [94, 95]. Huenecke et al. [96] investigated the reconstitution of NK cell populations following hematopoietic stem cell transplantation (HSCT) and found that, after 12 months, their distribution matched the 50th percentile of the reference range for healthy individuals, while patients suffering from acute and chronic GvHD were characterized by a delayed reconstitution of NK cells. The study further found that the CD56^bright^ subset was the first to appear posttransplantation, with CD56^int^ NK cells 3 months post-HSCT, followed by CD56^dim^ cells. CD56^bright^ and CD56^int^ shared almost identical expression marker profiles, unlike CD56^dim^ cells which lacked KIRs, CD62L, NKG2A, and CD57. While the presence of elevated levels of CD56^bright^ cells correlated to patients lacking GvHD, the identification of CD56^bright^ cells as a potential prognostic factor for GvHD requires further studies, not least because of confounding factors including, among others, patient data reproducibility and GvHD pathology grade.

## 5. Strategies for the Genetic Modification of NK Cells

For more than a decade, investigators have pursued methods to genetically engineer NK cells for use in clinical therapy against cancer. One of the earliest examples of genetic modification of NK cells involved the retroviral transduction of the cells with IL-2 cDNA to induce expression of endogenous IL-2 and enhance the cells' persistence *in vivo* [97] and reduce the cells' reliance on exogenous supply of cytokines. Since then, genetic modification of NK cells has expanded to include a number of additional approaches: from enhancing their target recognition specificities by the engineering of CARs to suppressing tumor microenvironment inhibition. Engineering of NK cells has most commonly been achieved with viral vectors, delivering a variety of transgenes, with CARs being among the most common ones. These are fusion proteins composed of an antigen recognition domain―typically an antibody single-chain variable fragment (scFv)―fused to a variety of intracellular signaling domains. The second and third generation CARs, wherein the extracellular antigen recognition domain is fused to two or three transmembrane and intracellular signaling domains, have been most commonly developed with NK cells (Table 2). Strategies aimed at improved NK cell immunotherapy through genetic engineering have enabled the enhancement of persistence, safety, and efficacy (Table 2). Persistence has been achieved via the engineering of cytokine-expressing NK cells, which can retain sustained IL-2 and IL-15 self-expression during their effector function. Efficacy has been achieved via the engineering of CARs targeting and improving the recognition of cancer-associated antigens, while the use of allogeneic sources of NK cells, such as NK cell lines, or incorporation of suicide genes—namely, inducible caspase 9 (iCasp9)—has been aimed at improving safety.

One of the main challenges associated with engineering NK cells is the low gene transfer efficiency of blood NK cells with either viral-based or nonviral methods, though viral-based transduction has been associated with significantly higher yields. Other issues being explored for overall improvement include efficiency of vector-mediated intracellular trafficking of nucleic acid cargo, immune response, regulatory issues that relate to manufacturing and standardization, and commercialization. Improvements in vector design as well as the use of new biomaterials as nucleic acid carriers are being pursued to enhance safety while improving gene transfer efficiency.

### 5.1. Viral Vectors for Genetic Engineering of NK Cells

Though difficult to transduct, efficiency of transduction of NK cells is relatively high with viral vectors compared to nonviral methods. The most commonly used viral vectors for the generation of CAR-engineered immune cells are part of the retrovirus family which includes *α*-, *β*-, *γ*-, *δ*-, and *ε*-retroviruses, spumaviruses, and lentiviruses. Integration profiles have been defined for all except *ε*-retroviruses [98]. During retroviral genome integration, the viral RNA genome, reverse transcribed into double-stranded DNA, associates to the host cell chromatin and integrates in the genome via the activity of integrase, a viral protein encoded by the *pol* gene. Based on their integration profiles, retroviruses can be divided into three groups. These include, firstly, murine leukemia virus (MLV), foamy virus (FV), and human T cell leukemia virus (HTLV), secondly, human immunodeficiency virus (HIV) and simian immunodeficiency virus (SIV), and, thirdly, avian sarcoma-leucosis virus (ASLV), which is the least discriminatory in terms of integration preferences. Among these, lentiviral or *α*-retroviral vectors are most commonly used [99, 100]. Like all retroviruses, they allow for stable integration with prolonged expression of the desired transgene. While new generations of vectors have shown significantly improved safety, toxicity and immunogenicity are still being cited as potential drawbacks to their clinical use [101, 102]. During transduction of an NK cell, the gene-encoding vector is semirandomly and stably incorporated into the host NK genome. This can result in insertional mutagenesis and has been reported in a number of studies using *γ*-retroviral vectors for the treatment of a variety of pathologies, including X-linked severe combined immunodeficiency and the Wiskott-Aldrich syndrome [103–106]. It is considered one of the main drawbacks to using viruses as gene transduction vectors. Since the beginning of the use of viral vectors in gene therapy, vector design approaches have employed architectures aimed at driving improved transgene expression, such as the incorporation of long terminal repeats (LTRs) [107]. These are homologous regions flanking the 5′- and 3′-termini of the double-stranded proviral DNA genome. These regions code for DNA that mediates circle formation prior to integration of the proviral DNA into the host cell. Additionally, the use of a self-inactivating sequence (SIN) adapted from lentiviral vectors to *γ*- and *α*-retroviral vectors enables the elimination of promoter/transcriptional activity of the wild-type LTR to address concerns regarding insertional mutagenesis [108]. Moreover, reducing the likelihood of replication competent retroviruses (RCRs) being formed has been achieved by a number of strategies including deletion of genes required for self-replication and reduction of overlapping sequences of viral genomes and vector-producing cells [109]. These strategies have been used in a number of clinical studies [110]. Insertional mutagenesis has been a recognized safety issue with *γ*-retroviral vectors. The use of integrase-deficient lentiviral vectors has demonstrated potential safety improvements [111]. These vectors have been used preclinically in cancer therapy [112]. They have gene packaging capability that is comparable to integrating lentiviral vectors and are expressed transiently with limited integration capability, thus enhancing safety by avoiding genomic integration. As a result, they are primarily used for the transduction of postmitotic cells. Due to the transient nature of gene expression mediated by these vectors, they suffer from the drawback of loss of gene expression in dividing cells as nonviral vectors. Though the clinical safety of *γ*-retroviral vectors with transcriptionally active LTRs has raised concerns [113], recent reviews reflect a robust safety pattern [114]. Suicide gene systems [115, 116] have also been used within the context of enhanced safety of long-living virally engineered immune cells. With NK cells having a relatively short *in vivo* persistence, the importance of suicide genes has not yet been extensively studied in these cells.

*γ*-Retroviral vectors were among the first vectors to be used for transduction and in gene therapy, while *α*-retroviruses entered the field somewhat later. They generate a polyclonal transduced population and have a slightly larger wild-type genome size compared to that of lentiviral vectors. The first examples involved genetically engineering NK cells to express IL-2 and avoid endogenous addition of cytokine following adoptive transfer [117]. Though the efficiencies were low (<5%), slight improvement was obtained with cell lines such as NK-92 [118]. A possible explanation for NK cells' resistance to retroviral transduction may stem from the cells' inherent defense mechanisms against viral infection, having ultimately evolved NK cells to reject exogenous gene transfer [119]. A strategy to improve retroviral transduction efficiency involved preactivating NK cells with both cytokine (IL-2) and K562 cells engineered to express membrane-bound IL-21 [120]. This resulted in successful transduction of immature NK cells, while nonactivated and highly mature CD57^+^ NK cells remained nontransduced. Retroviral transduction of NK cells requires rapidly diving cells and is thus most efficient with cells that have been expanded ex vivo [121]. In addition, multiple rounds of transduction are often needed. Guven et al. [122] obtained an increase in retroviral transduction of NK cells from 27% and 52% (after one round) or 47% to 71% (after two rounds) without expansion or following 21 days of ex vivo expansion, respectively. NK-92 cells can now be transduced with all retroviral vectors with high efficiencies, and transduction efficiencies from 60% to above 90% can now routinely be obtained with *γ*-retroviral vectors [123].

Lentiviral vectors, unlike their other retroviral counterparts, do not require actively dividing cells for effective transduction [124], as their preintegration complex is actively transported into the nucleus during the interphase. They contain a set of accessory genes the products of which are involved in the regulation of transcription. Their typical titer is higher than that of retroviral vectors (10^7^–10^8^ IFU/ml compared to 10^6^ IFU/ml for retroviruses). While lentiviruses are capable of genome integration, integrase-deficient versions of these have been developed. It is important to mention, however, that integrase-deficient variants can be developed for all retroviruses. The use of lentiviral vectors with NK cells has, however, been challenging. Standardized protocols have reported lentiviral transduction efficiencies from 15% for NK-92 cells to 30–40% for LNK, YT, and DERL7 cell lines [125]. For primary NK cells, stimulation with IL-2 and IL-12 was shown to enhance transduction efficiency by up to fivefold [126]. However, Tuli et al. reported diminished lentiviral transduction efficiency after 7 days of NK cell expansion [127] due to reduced NK viability, indicating that expansion conditions are important for maintaining NK cells' amenability to being transduced.

Boissel et al. [128] reported that, compared to mRNA transfection of anti-CD19 and anti-CD20 CARs, lentiviral transfection yields superior transgene expression, up to 73% when cord blood-derived NK cells were used, compared to <10% for mRNA transfection. For peripheral blood, efficiency was lower, however, and did not exceed 16%.

The use of reagents such as polybrene, protamine sulfate, or Retronectin has been employed to enhance the efficiency of viral transduction of NK cells. Polybrene and promatine sulfate, both cationic polymers, work by reducing NK cell virus repulsion [129], thereby increasing the efficiency of nucleic acid-membrane fusion. Polybrene has been used routinely in the transduction of genes, including CARs, into primary NK cells [80, 130–132], while its use with NK-92 cells has been limited due to its reported toxicity with that cell line. DEAE-dextran and poly-L-lysine have also been used [133].

Retronectin, on the other hand, is a truncated version of the extracellular matrix cell adhesion protein fibronectin and works by supporting colocalization of viral particles within its binding domains [134]. Lowe et al. [135] described a strategy to enhance lentiviral transduction of second generation CARs into NK cells derived from gene-modified hematopoietic stem cells using Retronectin CH-296. Vectofusin-1, an alternative transduction enhancer, was recently reported as a potential alternative to Retronectin [136]. Denning et al. [137] tested a variety of polycations and determined that, depending on the specifics of the cell population, no cation can be claimed to be superior at enhancing viral transduction, and studies should test multiple variants to determine optimal performance.

Efforts at altering the host range of retroviral vectors―also known as pseudotyping, which results in the incorporation, on viral vectors, of glycoproteins from other enveloped viruses―have been also extensively studied [138]. Common methodologies for viral pseudotyping have been described at length [139], alongside the pseudotyping of emerging viruses [140]. Most commonly, pseudotyping has been carried out with vesicular stomatitis virus GP (VSV-G). This is because of its broad tropism and stability of the resulting pseudotypes. VSV-G-pseudotyped lentiviral vectors were shown to enhance transduction of NK cells in association with BX795, an inhibitor of the TBK1/IKK*ɛ*, collectively weakening antiviral NK cell responses thus enhancing gene transfer [141]. However, further clarity on the use of VSV-G-pseudotyped vectors with NK cells is needed.

### 5.2. Nonviral NK Cell Engineering

In large part due to the safety concerns associated with the use of viral vectors, nonviral alternatives have seen a significant increase between the early 2000s and now. These include all synthetic gene carriers that can be used to effect stable gene expression in target immune cells. A major characteristic of most nonviral gene carriers is their induction of transient gene expression. The nature of gene delivery from polymers or liposomes bearing cationic lipids is biphasic, characterized by transient expression that lasts a few days followed by prolonged, albeit lower level expression. The transgene, initially in the nucleus, is thought to eventually be lost away with the dividing cells [142, 143]. Though inducing genomic integration with nonviral carriers can be achieved with sleeping beauty or piggyBac systems, no substantial examples with NK cells yet exist of these approaches beyond a handful of preliminary reports (Table 2). PiggyBac transposon systems operate by recognition of transposon-specific inverted terminal repeat sequences on both ends of the transposon vector by a transposase. Upon recognition, the transposase integrates genetic elements from the original sites into chromosomal sites. This creates stable genomic integration of target genomic material. They have most commonly been used for genetic modification of T cells [144]. Transient gene expression may be viewed favorably when working with new carriers or transgenes whose safety profile has not been established. It also plays to the relatively short-term persistence of NK cells *in vivo*, which is under two weeks, associating a longer-term response with the need for repeated infusions of transfected NK cells. However, safety profiles for many of the emerging nonviral vectors are yet to be established.

#### 5.2.1. Electroporation and Nucleofection

One of the earliest strategies for nonviral gene transfer into NK cells has been via electroporation. Electroporation-based methods for transfection of NK cells are considered safer than viral-based methods due to the transient nature of gene expression and avoidance of genomic integration of foreign genetic material which could result in unwanted replication and, as such, prone to fewer regulatory constraints. Electroporation is based on the generation of an electric field to induce temporary permeabilization of the cell membrane. While well tolerated, cell damage due to irreversible electroporation can be a concern [145]. Liu et al. [146] demonstrated successful restoration of lytic function to NK cell line YT-1(−) lacking expression of CD11a/CD18 following transfection of CD18 via electroporation. The authors identified that CD18 was responsible for the loss of cytotoxic function in YT-1(−) cells―defective in CD18 at the transcriptional and protein levels―and showed that transfection with a plasmid-bearing CD18 could partially restore CD11a/CD18 expression. Following transfection, CD11a/CD18 surface expression was detected in 12.8/11.9% to over 99% of cells without selection. Since these early studies, electroporation has been adopted in a large number of NK transfection studies, with routine transfection efficiencies of above 50%. Commercial, GMP-compliant electroporation equipment is now also available [147]―MaxCyte is developing a number of clinical-grade platforms that can support GMP-compliant electroporation of cells at high efficiencies, while Miltenyi has developed a closed-system electroporator as part of their CliniMACS line of equipment. Using one of these systems, Carlsten et al. [148, 149] described a GMP-compliant process for electroporation of mRNA coding for the chemokine receptor CCR7 and the antibody-binding receptor CD16 (CD16-158V). Improved NK cell homing and cytotoxicity against lymphoma cells were achieved, alongside close to 100% transgene expression. Similarly, anti-CD19 CARs were also successfully electroporated into peripheral blood-derived NK cells with an efficiency of up to 81% [150]. Recently, engineered microsystems for so-called nanoelectroporation have been described which have enabled, driven by dielectrophoresis, stable transfection of anti-CS1 CAR genes in up to 60,000 NK cells/cm^2^.

While electroporation requires dividing cells in the exponential growth phase, nucleofection was developed to achieve gene transfer to the nucleus without the need for cell division. When applied to NK cells [151], superior transfection efficiencies of over 50% were achieved in early studies. Nucleofection has since been used to efficiently engineer NK cells with a range of CARs, such as anti-ROR for targeting metastatic solid tumors [152] or anti-CD20 for targeting B-cell non-Hodgkin lymphoma [153] or Burkitt lymphoma [154]. Alongside engineering NK cells to express CARs, a promising strategy to immunotherapeutic interventions has been through the silencing of genes that cause the suppression of effector functions of NK cells. One such target is transforming growth factor-beta (TGF-*β*), a potent immunosuppressor that has a negative impact on surrounding NK cells in the tumor microenvironment. The levels of TGF-*β* are often elevated in the serum of cancer patients, and this is associated with weakened NK cell responses. Zhao et al. [155] reported nucleofection of NK-92 cells with pTAR-GET plasmid expressing dominant-negative TGF-*β* type II receptor (DNT*β*RII), which blocks the TGF-*β* signaling pathway and restores the killing ability of NK cells. By blocking TGF-*β* signaling, detected through the lack of phosphorylation of Smad-2 and Smad-3 in genetically modified NK cells, the authors observed restored lytic function of NK cells against MCF-7 breast cancer cells. Elsewhere, techniques for siRNA-mediated knockdown of gene expression of NK cells via nucleofection using patented technologies such as ON-TARGETplus SMART poolsiRNA have also been described [156].

Electroporation has also been used to generate gene-edited NK cells *in vitro* via clustered regularly interspaced short palindromic repeat (CRISPR)/CRISPR-associated (Cas9). Dong et al. [157] created tumor suppressor gene *PRDM1*-modified NK cells as models of natural killer cell lymphoma and have shown that gene editing of NK cells can be a powerful approach to study functional alterations in human tumor suppressor genes.

It is important to point out that, although electroporation has been successfully demonstrated as an effective approach for the genetic modification of NK cells, considerations in regard to the type of gene modification and the expected application are critical. Due to the nature of electroporation and nucleofection, stable genomic integration of DNA is not achievable with regular plasmid DNA or RNA transfection. For these reasons, electroporation can be combined with the transfection of genes that code for the expression of cytokines or other stimulatory factors to enhance sustained persistence of cells, especially in solid tumor environments where longer-term responses and enhanced persistence are more critical.

#### 5.2.2. Trogocytosis

Trogocytosis is the transfer of membrane patches from antigen-presenting cells by lymphocytes through an immune synapse, followed by subsequent expression of these molecules on the lymphocytes' own surface [158]. This process has been employed to engineer the expression of specific molecules on the surface of NK cells for enhanced cytotoxic function. Trogocytosis-mediated transfer of the chemokine receptor CCR7 was designed to occur from engineered K562 cells to the surface of human NK cells, resulting, in a study by Somanchi et al. [159], in CCR7 expression in 80% of NK cells following 1 h coculture, alongside enhanced lymph node migration. Elsewhere, transfer of CCR7 to NK cells was also demonstrated following coculture with mature dendritic cells (mDCs) or Epstein-Barr virus- (EBV-) transformed lymphoblastoid cell lines [160].

More recently, trogocytosis was also employed to effect expression of CARs on the NK cell surface. Cho et al. [161] engineered the expression of anti-CD19-BB-*ζ* on K562 cells, which were then cocultured in the presence of peripheral blood-derived CD56^+^CD3^−^ NK cells. Trogocytosis resulted in acquisition of the anti-CD19 CAR in 18.6% of NK cells following 1 h of coculture, followed by enhanced cytotoxicity of NK cells against multiple B-ALL cell lines. However, trogocytosis is characterized by relatively short gene transfer times (72 hours for CCR7 and 2 h for CD19) and has so far relied on liver donor cells to reach high expression levels (from 50% to 80% for CCR7 and 19% to 47% for CD19). This significantly limits the clinical outlook of this technology and places it at the fringes of current approaches for nonviral gene transfer.

#### 5.2.3. Polymer, Cationic Lipid, and Nanoparticle-Based Transfection

While viral transduction and electroporation-based approaches have resulted in high efficiencies of gene expression in NK cells, these methods can be laborious and require specialized equipment and laboratory setups. For that reason, nonviral transfection approaches that utilize nanoparticles, liposomes, or polymers as nucleic acid carriers have also been under investigation. As an emerging area of investigation, these studies are still largely preliminary, and optimization of material properties greatly affects transfection outcome.

Lipofection―a cationic liposome-based transfection technique―has been sparingly used to transfect primary NK cells, largely due to relatively low transfection efficiencies associated with it. One of the earliest studies on cationic liposome-medicated transfection demonstrated transfer of IL-2-expressing plasmid into primary NK cells using DMRIE/DOPE liposomes [162]. More recently, commercial lipofection reagent Lipofectamine 3000® was used to transfect primary NK cells with tumor suppressor microRNA miR-27a-5p inhibitor in a study by Regis et al. [163]. An efficiency of transfection, based on detection of miRNA in target cells following electroporation, of 30% was achieved, while the authors showed that miR-27a-5p directly modulates the expression of CX_3_CR1. Elsewhere, Youness [164] described lipofection of miR-486-5p into primary human NK cells to target hepatocellular carcinoma. miR-486-5p was recognized as a direct regulator of insulin-like growth factor-1 receptor (IGF-1R), a modulator of hepatocellular carcinoma. Following lipofection, elevated expression of NKG2D and perforins was recorded. By using a combination of miRNA electroporation and siRNA knockdown of IGF-1R, the authors showed that miR-486-5p acts by both enhancing NK cell cytotoxicity through elevated NKG2D and perforin expression and supporting tumor progression by modulating members of the IGF axis.

Unlike lipofection which is based on the use of cationic lipids, nanoparticles have been assembled from a variety of materials. Nanoparticles are synthetic engineered particles that range in size from 1 to a few hundreds of nm. They behave autonomously, are able to be synthesized easily in the laboratory, and can be designed to encapsulate and deliver genetic material [165]. Though widely used in drug delivery [166], the use of nanoparticles for the engineering of NK cells is still in its infancy. A multifunctional lipid nanoparticle containing the lipid YSK12-C4, cholesterol, and PEG-DMG, called YSK12-MEND, was developed to effect delivery of GAPDH siRNA to immune cells [167]. When transfected with the nanoparticles, NK-92 cells yielded 75% transfection efficiency, lower than that obtained with Jurkat, THP-1, or KG-1 cells, but higher than many lipid-based transfection systems, including Lipofectamine® RNAiMAX, which the authors tested and which yielded about 19% transfection efficiency. To achieve these responses, the authors used doses of nanoparticle between 1 and 30 nM. This was likely, the authors argued, due to low aggregability of the nanoparticles, thereby enhancing their accessibility to the cells in the medium. However, the manufacturing procedure of YSK12-MEND is more laborious compared to off-the-shelf alternatives and further optimization of performance characteristics for primary cells needs to be made. Currently, all data is based on cell lines which are considerably easier to transfect.

By comparing the gene transfer efficiency of Lipofectamine 2000®, polyethylenimine (PEI), and magnetic iron oxide nanoparticles into peripheral blood mononuclear cells, Przybylski et al. [168] found that there were strong effects on the proliferative ability of immune cells induced by the various nanoparticle systems. In particular, iron oxide nanoparticle exerted a strong antiproliferative effect on immune cells both *in vitro* as well as *in vivo*, in contrast to Lipofectamine, which enhanced proliferation, or PEI which inhibited proliferation *in vitro* but showed increased mouse survival *in vivo*. Overall, these results have suggested that various nanoparticle systems act by mediating specific pro- or anti-inflammatory mechanisms which might have broader immune effects, so their choice might require considerations of specific effects that can be induced by their use.

Nanoparticles are also being used to enhance expansion of NK cells for adoptive transfer, independent of direct transfection. Oyer et al. [169] developed one such nanoparticle-based technology, which is based on the use of particles prepared from K562-mb21-41BBL cells, which express 41BBL and membrane-bound interleukin-21. When these particles were used to stimulate NK cells *ex vivo* alongside low-dose interleukin-2 (1000 U/three times per week), the authors reported a 66-fold higher amount of NK cells. This technology is currently proceeding to clinical trials.

Other polymers and transfection reagents are increasingly being utilized for the genetic modification of primary immune cells, most notably T cells. It is expected that as these approaches mature, they will be applied to the genetic engineering of NK cells in place of viral- or electroporation-based methods. As a whole, nanoparticle-based approaches for gene transfer effectively address issues of safety, biocompatibility, and immunological control. On the other hand, few if any have successfully demonstrated comparable performance to viral-based approaches. Moreover, nanoparticle-mediated gene transfer necessitates a cascade of extra- and intracellular events to take place to traffick the transgene of interest across the cell, out of the endosome and into the nucleus, placing additional design constraints on their development. To support the intracellular delivery of genetic cargo, nuclear localization signaling peptides [170] and microtubule-associating sequences [171] have been incorporated into nonviral gene delivery carriers; however, their use is still highly experimental, indicating that significantly more optimization is needed before the fully fledged use of these systems can take off.

## 6. Clinical Perspective and Outlook

Despite the extensive body of work, only a handful of NK-based therapies have progressed to the clinic. In the United States, two clinical trials (NCT00995137 and NCT03056339) with genetically modified NK cells are recorded as of mid-2018, both redirected against CD19 using CARs. There are a total of eight clinical trials registered worldwide utilizing genetically engineered NK cells, two of which are targeting solid tumor malignancies (Table 3). These trials are driving home the promise of NK cells as safer alternatives to engineered T cells. However, as discussed above, multiple issues exist that need to be considered when designing adoptive NK cell-based cancer immunotherapies: efficacy and safety being the primary―though by no means only―ones. While not all issues that have been associated with CAR-T cells will apply to NK cells, such as induction of GvHD, a more comprehensive understanding of the importance of safety mechanisms such as the introduction of suicide genes to NK cell therapies is needed.

As these trials approach more advanced stages and the dose capacity increases, issues related to manufacturability will become more prominent. While remarkable advances have been made with the isolation, expansion, and manipulation of these cells―in one of the clinical trials, for instance, NK cells are coexpanded in the presence of K562-mb15-41BBL cells―scaling up production is still a major challenge. Companies such as Cyto-SEN are set to embark on phase I clinical trials with adoptively transferred NK cells based on novel, nanoparticle-based approaches for expanding these cells. Not unlike T cell-based immunotherapies, most preclinical and clinical success with engineered NK cells has so far been observed with hematological malignancies. The use of NK cells to target solid tumors suffers from deep TME immunosuppression due to metabolic reprogramming, tumor heterogeneity, and hypoxia, resulting in poor tumor infiltration of NK cells and, ultimately, poor antitumor immunity [172]. However, an ongoing clinical trial against MUC-1^+^ solid tumors and one targeting metastatic solid tumors responsive to NKG2D (Table 3), alongside advancements in improving the trafficking of NK cells into solid tumors by engineering chemokine receptors such as CXCR2 [173, 174] into solid tumors, are demonstrating advancements into targeting cancers that have traditionally been difficult to treat with NK cells.

For the successful translation of genetically modified NK cells, issues of vector safety and efficacy will have to comply with regulatory guidelines. The International Organization for Standardization (ISO) has begun development of cell therapy standards, with the first one set for publication in 2018, while the FDA announced a comprehensive regenerative medicine policy framework in November 2017, which highlights safety and efficacy in particular. Manufacturing of engineered NK cells for clinical trials is currently carried out in GMP facilities under decentralized manufacturing models, with third-party vector engineering labs providing vectors for genetic fusion with NK cells. At the same time, development of cGMP-compliant electroporation equipment continues, which is enabling the testing of point-of-care manufacturing that not only includes cell isolation and proliferation but also genetic modification steps, for the better deployment of engineered cell therapies.

It is evident that the increasing variety and number of preclinical investigations with genetically engineered NK cells are poised to lead to an expansion in clinical-stage studies. However, successful clinical outcomes are going to depend on the convergence of vector engineering and manufacturing, cell culture enhancements, understanding of NK cell biology, and compliance with maturing regulatory frameworks.

## Figures and Tables

**Table 1:** Differences between CAR-NK and CAR-T cells.

	CAR-NK cells	CAR-T cells
Sources	Cord blood, peripheral blood, iPSC, cell lines	Cord blood, peripheral blood, iPSC
Expansion	Flasks or bag-based expansion systems with cytokines (IL-2, IL-12, IL-15, IL-18, IL-21); or feeder cell lines (engineered K562 cells)	Flasks or bag-based expansion systems with cytokines (IL-2 or IL-7)
Use	Autologous; allogeneic possible	Autologous; allogeneic with MHC match
Engineering methods	Viral transduction, electroporation/nucleofection, nanoparticles, trogocytosis	Viral transduction, electroporation/nucleofection, nanoparticles, trogocytosis
Transfection efficiencies	Low even with viral vectors	Higher than for NK cells
Adoptive transfer considerations	Limited persistence	GvHD
		Cytokine storm
		Suicide genes needed

**Table:** (a) Enhancing persistence

Gene	Gene transfer vector	Cell type	Target	Gene transfer efficiency	Ref
IL-2	Retrovirus	Peripheral blood NK cells	K562 and Raji cells (*in vitro*)	N/A	Miller et al. [97]
IL-2	Retrovirus	NK-92 and YT	Liver metastasis	10–20%	Nagashima et al. [118]
IL-15	VSV-G-pseudotyped lentivirus	NK-92 and NKL	Breast carcinoma (*in vitro*)	4%	Sahm et al. [175]
IL-15	Retrovirus	NK-92	Leukemia, lymphoma, and solid tumor cells (*in vitro*); leukemia and sarcoma cells (*in vivo*)	71% (range 23%–97%)	Imamura et al. [176]
IL-15	Electroporation	NKL	Human hepatoma cells (*in vitro*)	N/A	Jiang et al. [177]

**Table:** (b) Chimeric antigen receptors

Gene	Gene transfer vector	Signaling components	Cell type	Target	Gene transfer efficiency	Ref
CD19	mRNA electroporation	CD3*ζ* and 4-1BB	Primary NK cells	B cell malignancies	>95% cells	Carlsten et al. [178]
EGFR	Lentivirus	CD3*ζ* and CD28	NK-92 and NKL	Glioblastoma	N/A	Han et al. [80]
CD19, antimesothelin	*Sleeping beauty* transposon	CD3*ζ*, CD28, and 4-1BB	iPSC-derived NK cells	B cell malignancies, ovarian cancer	N/A	Ni et al. [179]
CD19 and IL-15 with iC9 suicide gene	Retrovirus	CD3*ζ* and CD28	Cord blood NK cells	B cell malignancies	>80%	Liu et al. [66]
ROR1	mRNA electroporation	4-1BB	Peripheral blood NK cells	Neuroblastoma and sarcoma	70% cells	Park et al. [180]
CD22 and CD19-ENG	Retrovirus	CD28 and 4-1BB	Peripheral blood NK cells	B cell malignancies	70–80% of CD22-CARs, and ~50% bispecific CD19-T cell engagers	Velasquez et al. [181]
CD19	VGV-pseudotyped retrovirus	CD28-CD3*ζ* or CD137-CD3*ζ*	NK-92	B cell malignancies	N/A	Oelsner et al. [182]
CD123	*α*-Retroviral SIN	CD28 and 4-1BB	Peripheral blood NK cells	Acute myeloid leukemia	22.9% on day 3; 11.9% on day 9	Klöß et al. [183]
CD20	pLXSN retrovirus	CD3*ζ*	NK-92	B cell malignancies (*in vitro*)	93.8–96.3%	Müller et al. [184]
CS1	PCDH lentivirus	CD3*ζ* and CD28	NK-92 and NKL	Multiple myeloma	>98% (NK-92), >95% (NKL)	Chu et al. [185]
CD4	Lentivirus	CD3*ζ*, CD28, and 4-1BB	NK-92	T cell lymphoma	>85%	Pinz et al. [186]
PSCA	Self-inactivating pHATtrick lentivirus	CD3*ζ*, CD28, and DAP12	Peripheral blood NK cells	Prostate cancer stem cells	~50%	Töpfer et al. [74]
NKp44	Retrovirus	CD3*ζ* and CD28	Peripheral blood NK cells	Enhanced cytotoxicity	N/A	Kasahara et al. [187]
CD19 and HER2	pCCW lentivirus	CD3*ζ*, CD28, and 4-1BB	NK-92	Solid tumors	N/A	Siegler et al. [188]
CD20	mRNA nucleofection	CD3*ζ* and 4-1BB	Peripheral blood NK cells	Pediatric Burkitt lymphoma	N/A	Chu et al. [189, 190]
CD138	Lentivirus	CD3*ζ* (with CD8*α*)	NK-92MI	Multiple myeloma	>95%	Jiang et al. [130]
ErbB2	Lentivirus	CD3*ζ* and CD28	NK-92	Glioblastoma	N/A	Steinbach et al. [191]
GD2	pLXSN retrovirus	CD3*ζ*	NK-92	Neuroblastoma	N/A	Esser et al. [192]
CD33	Electroporation	CD3*ζ*	YT	Acute myeloid leukemia	90% (after enrichment)	Schirrmann and Pecher [193]
NKG2D	Retrovirus	CD3*ζ* and DAP10	Peripheral blood NK cells	T-cell ALL, B-cell ALL, osteosarcoma, prostate carcinoma, rhabdomyosarcoma, neuroblastoma, Ewing sarcoma, colon carcinoma, gastric carcinoma, lung squamous cell carcinoma, hepatoma, and breast carcinoma (*in vitro*), osteosarcoma (*in vivo*)	80% (range 67–96%)	Chang et al. [194]
EGFR	Lentivirus	CD3*ζ* and CD28	NK-92	Breast cancer	39.4%	Chen et al. [195]
HER-2	Retrovirus	CD3*ζ* and CD28	Peripheral blood NK cells	Ovarian and breast cancer cell lines	55 ± 11%	Kruschinski et al. [131]
CD19	Trogocytosis	CD3*ζ* and 4-1BB	Peripheral blood NK cells	B-ALL cell lines	47% upon coculture with donor cells	Cho et al. [161]
TRAIL-receptor 1	Retrovirus	CD3*ζ*, 4-1BB, and CD28	KHYG-1	Colo205, Daudi, K562 cells (*in vitro*)	N/A	Kobayashi et al. [196]
GPC3	Lentivirus	CD8, CD28, and CD3*ζ*	NK-92 and peripheral blood NK cells	Hepatocellular carcinoma	~35% (primary NK cells)	Yu et al. [197]

**Table:** (c) Enhancing cytotoxicity

Gene	Gene transfer vector	Cell type	Target	Gene transfer efficiency	Ref
miR-27a-5p miRNA	Lipofection	Peripheral blood NK cells	Enhanced cytotoxicity	N/A	Regis et al. [163]
DNT*β*RII	Retrovirus	Cord blood NK cells	Enhanced cytotoxicity	75.8%	Yvon et al. [198]
DNT*β*RII	Nucleofection	NK-92	Enhanced cytotoxicity	N/A	Zhang et al. [155]
CD16 cDNA	Retrovirus	NK-92	Antibody-dependent cell-mediated cytotoxicity	N/A	Binyamin et al. [199]

GALV: gibbon ape leukemia virus. All studies present data *in vivo* unless otherwise stated.

**Table 3:** Registered worldwide clinical trials with genetically engineered NK cells.

Target	Sponsor and Clinicaltrials.gov identifier	Disease	Phase	Cell type
CD7—TCR*ζ*, CD28, and 4-1BB	PersonGen BioTherapeutics, Suzhou, Jiangsu, China NCT02742727	Leukemia and lymphoma	Phase I/II	NK-92 cells
CD19/TCR*ζ*/CD28 and 4-1BB	PersonGen BioTherapeutics, Suzhou, Jiangsu, China NCT02892695	Relapsed/refractory ALL, CLL, FL, BCL, DLBCL	Phase I/II	NK-92 cells
CD33/CD28/4-1BB	PersonGen BioTherapeutics, Suzhou, Jiangsu, China NCT02944162	Relapsed/refractory ALL	Phase I/II	NK-92 cells
MUC1	PersonGen BioTherapeutics, Suzhou, Jiangsu, China NCT02839954	MUC1^+^ solid tumors	Phase I/II	Peripheral blood NK cells
CD19/4-1BB/CD18/iCasp9/IL-15	MD Anderson Cancer Center, Houston, TX, USA NCT03056339	B cell malignancies	Phase I/II	Cord blood-derived NK cells
CD19/4-1BB/CD3*ζ*	National University of Singapore, Singapore NCT01974479	B-cell ALL	Phase I	Peripheral blood NK cells
CD19/4-1BB/CD3*ζ*	St. Jude's Children Research Hospital, Memphis, TN, USA NCT00995137	ALL	Phase I	Peripheral blood NK cells
NKG2D	The Third Affiliated Hospital of Guangzhou Medical University NCT03415100	Metastatic solid tumors	Phase I	Peripheral blood NK cells

ALL: acute lymphoblastic leukemia; CLL: chronic lymphocytic leukemia; FL: follicular lymphoma; BCL: B cell lymphoma; MCL: mantle cell lymphoma; DLBCL: diffuse large cell lymphoma; AML: acute myeloid leukemia; NHL: non-Hodgkin Lymphoma.
